# Tuning of the Lethal Response to Multiple Stressors with a Single-Site Mutation during Clinical Infection by *Staphylococcus aureus*

**DOI:** 10.1128/mBio.01476-17

**Published:** 2017-10-24

**Authors:** Krishan Kumar, John Chen, Karl Drlica, Bo Shopsin

**Affiliations:** a Departments of Medicine and Microbiology, New York University School of Medicine, New York, New York, USA; b Department of Microbiology and Immunology, Yong Loo Lin School of Medicine, National University of Singapore, Singapore; c Public Health Research Institute and Department of Microbiology, Biochemistry and Molecular Genetics, New Jersey Medical School, Rutgers Biomedical and Health Sciences, Newark, New Jersey, USA; Harvard Medical School

**Keywords:** Antibiotics, *Staphylococcus aureus*, *agr*, ciprofloxacin, daptomycin, gentamicin, resistance, tolerance

## Abstract

The *agr* system of *Staphylococcus aureus* promotes invasion of host tissues, and as expected, agents that block *agr* quorum sensing have anti-infective properties. Paradoxically, *agr*-defective mutants are frequently recovered from patients, especially those persistently infected with *S. aureus*. We found that an *agr* deficiency increased survival of cultured bacteria during severe stress, such as treatment with gentamicin, ciprofloxacin, heat, or low pH. With daptomycin, deletion of *agr* decreased survival. Therefore, *agr* activity can be either detrimental or protective, depending on the type of lethal stress. Deletion of *agr* had no effect on the ability of the antimicrobials to block bacterial growth, indicating that *agr* effects are limited to lethal action. Thus, the effect of an *agr* deletion is on bacterial tolerance, not resistance. For gentamicin and daptomycin, activity can be altered by *agr*-regulated secreted factors. For ciprofloxacin, a detrimental function was downregulation of glutathione peroxidase (*bsaA*), an enzyme responsible for defense against oxidative stress. Deficiencies in *agr* and *bsaA* were epistatic for survival, consistent with *agr* having a destructive role mediated by reactive oxygen species. Enhanced susceptibility to lethal stress by wild-type *agr*, particularly antimicrobial stress, helps explain why inactivating mutations in *S. aureus agr* commonly occur in hospitalized patients during infection. Moreover, the *agr* quorum-sensing system of *S. aureus* provides a clinically relevant example in which a single-step change in the response to severe stress alters the evolutionary path of a pathogen during infection.

## IMPORTANCE

When phenotypes produced in response to an environmental stress are inadequate to buffer against that stress, changes that do buffer may become genetically encoded by natural selection. A clinically relevant example is seen with *S. aureus* mutants that are deficient in the key virulence regulator *agr*. Paradoxically, defects in *agr* are selected during serious hospital infection and have been associated with worse outcome. The current work helps resolve this paradox: *agr* mutants are often less readily killed by lethal stressors without affecting MIC, a phenomenon known as tolerance. Our results indicate that tolerance, which would not be detected as resistance, can be selected in clinical settings. The data also support the ideas that (i) *S. aureus* broadly hedges against environmental change and stress through genome plasticity, (ii) reactive oxygen can be involved in the self-destructive response in bacteria, and (iii) therapeutic targeting of *agr* and virulence can be counterproductive.

## INTRODUCTION

Experimental (1–3) and observational (4–7) work suggests that mutation of global regulators drives adaptive leaps made by microbes. In *Staphylococcus aureus*, the *agr* quorum-sensing transcriptional regulator is likely to be such a system. The *agr* regulon governs the expression of secreted virulence factors that appear to enhance acute infection and bacterial dissemination among healthy hosts. However, these factors are not needed, or are needed less, for pathogen persistence inside human tissues (8–12). Indeed, the prototypical within-host adaptation during *S. aureus* infection results in partial or complete inactivation of *agr* (13–17). While this regulator, which controls ~200 genes *in vitro* (18), is critical for pathogenesis in a variety of contexts (19–22), *agr*-defective mutants arise and are enriched during human infection when treated with antimicrobials (23–26). The result is poor clinical outcome (27, 28). Thus, the *S. aureus agr* system provides an opportunity to study how inactivation of a virulence regulon shifts the pathogen to a more persistent state. Moreover, understanding the enrichment of global regulator mutants during antimicrobial treatment is central to managing infections by *S. aureus* and other pathogens.

The *agr* locus consists of two divergent transcription units driven by promoters P2 and P3 (reviewed in reference 29). The P2 operon encodes the signaling module, which contains four genes—*agrB*, -*D*, -*C*, and -*A*—each of which is required for transcriptional activation of the *agr* regulon. AgrC is the receptor-histidine kinase, and AgrA is the response regulator. AgrD is the autoinducing, secreted peptide that is derived from a propeptide processed by AgrB. The P3 transcript is a regulatory RNA (RNAIII) that also contains the structural gene for delta-hemolysin. Regulation of target genes by *agr* occurs through two pathways: (i) an RNAIII-dependent regulation of virulence genes and (ii) an RNAIII-independent, AgrA-mediated regulation of metabolic genes and small cytolytic toxins known as phenol-soluble modulins (PSMs) (18). The regulatory connection between these processes links virulence to metabolism.

To study *agr*-stressor effects, it is important to distinguish growth-related phenotypes from those specific to survival. For example, treatment with an antimicrobial leads to damage that is specific to the test agent. This primary damage halts growth, which is measured as the MIC. The MIC reflects drug uptake, efflux, and target affinity; high MIC values are associated with antimicrobial resistance. Some forms of primary damage also kill cells, with much of the lethal process arising from a self-destructive, secondary bacterial response to the primary damage (reviewed in references 30 to 32). To focus experimental measurements on the lethal response, lethal drug concentrations are normalized to the MIC. It is also important to recognize that lethal stress may be transient. For example, reactive oxygen species (ROS) can accelerate killing without increasing the extent of killing (33). Consequently, overnight killing assays, such as those commonly used to measure minimal bactericidal concentration (MBC), may take too much time to detect changes in killing rate and may therefore be uninformative with respect to the stress response (33).

Highly lethal antimicrobials are important probes for studying bacterial responses to lethal stress, particularly for responses involving the accumulation of toxic ROS (30–32, 34–36). The present work used a range of both drug concentrations and treatment times to probe effects of *agr* status on the response of *S. aureus* to lethal stress. We found that an *agr* defect increased *S. aureus* survival by an order of magnitude following treatment with some antimicrobials (gentamicin) but that it conferred hyperlethality on others (daptomycin). *agr*-deficiency-mediated protection operated through a variety of mechanisms that depended on the underlying lethal stress. The data lead to a framework for interpreting the phenotypes of known and newly emergent infection-adaptive mutations. Broadly speaking, mutation can create, in a single step, complex traits that explain how loss of a seemingly important facilitator of virulence can be adaptive during infection by suppressing the lethal effects of stressors.

## RESULTS

### Transcription from *agr* promoters during growth in culture.

Because *agr* is a quorum-sensing regulon (29, 37), differences in antimicrobial-mediated killing between the wild type and *Δagr* mutants must be interpreted within the context of growth phase and *agr* induction. Using a β-lactamase reporter fused to the principal *agr* promoter, P3, we confirmed, in laboratory strain *S. aureus* Newman, that maximal *agr* activity is seen in late exponential growth phase and is followed by a plateau or decrease (see Fig. S1A in the supplemental material), as reported previously (38). All subsequent experiments were performed at late exponential phase.

10.1128/mBio.01476-17.1 FIG S1  Tests of *agr*P3 promoter activity. *S. aureus* Newman cells containing *agr*P3*-blaZ* (SaPI1 *attC*::*agr*P3-*blaZ*; strain BS983) were grown in TSB (A) or TSB in the presence of 20% (vol/vol) human serum (B). *agr*P3 activity (β-lactamase units/culture OD_600_) was assayed at the indicated times (see Materials and Methods). Symbols: broken line, growth; empty circles, *agr*P3 activity in TSB; filled circles, *agr*P3 activity in medium containing serum. Data represented the means from biological replicates ± standard deviations (*n* = 3). Download FIG S1, TIF file, 0.1 MB. Copyright © 2017 Kumar et al. 2017 Kumar et al. This content is distributed under the terms of the Creative Commons Attribution 4.0 International license.

### Survey of lethal agents affected by deletion of *agr*.

*agr*-defective strains have been associated with the development of vancomycin tolerance (39, 40); however, *agr* dysfunction is associated with only small reductions in killing that are apparent only with long-term, time-kill experiments (41, 42). Consequently, we focused on several agents known to be rapid, concentration-dependent killers of *S. aureus* (gentamicin, ciprofloxacin, and daptomycin). For these agents, a deficiency in *agr* (*agr*::*tetM* deletion) had no effect on bacteriostatic activity (MIC) compared with a wild-type strain (Table 1). Thus, *agr* has no effect on the initial, bacteriostatic lesion created by the drugs; lethal activity is likely a stress response to those lesions.

**TABLE 1:** Antimicrobial susceptibility^b^

Antibiotic or compound	Strain	TSB MIC (μg/ml for antibiotics, mM for compounds) for strain:		Serum MIC (μg/ml) for strain:	
		WT	Δ*agr* mutant	WT	Δ*agr* mutant
Antibiotics					
Cipro	Newman	0.5	0.5	1	1
	ATCC 25923	0.5	0.5	1	1
	LAC	0.5	0.5	1	1
Gent	Newman	6	6	12	12
	ATCC 25923	6	6	12	12
	LAC	6	6	12	12
	126a	3	3	6	6
	127b	3	ND	6	ND
	BS39	1.5	ND	3	ND
	BS40	1.5	ND	3	ND
Oxa	Newman	0.25	0.25	ND	ND
Dapto^a^	Newman	4	4	ND	ND
	LAC	2	2	ND	ND
Compounds					
H_2_O_2_	Newman	0.258	0.258		
2,2′-Bipyridyl	Newman	8	8		
Thiourea	Newman	400	400		

^a^ Daptomycin MICs were determined in Mueller-Hinton broth supplemented with 50 μg/ml Ca^2+^ and 25 μg/ml Mg^2+^.

^b^ Abbreviations: Cipro, ciprofloxacin; Dapto, daptomycin; Gent, gentamicin; ND, not determined; Oxa, oxacillin; WT, wild type.

**FIG 1:**
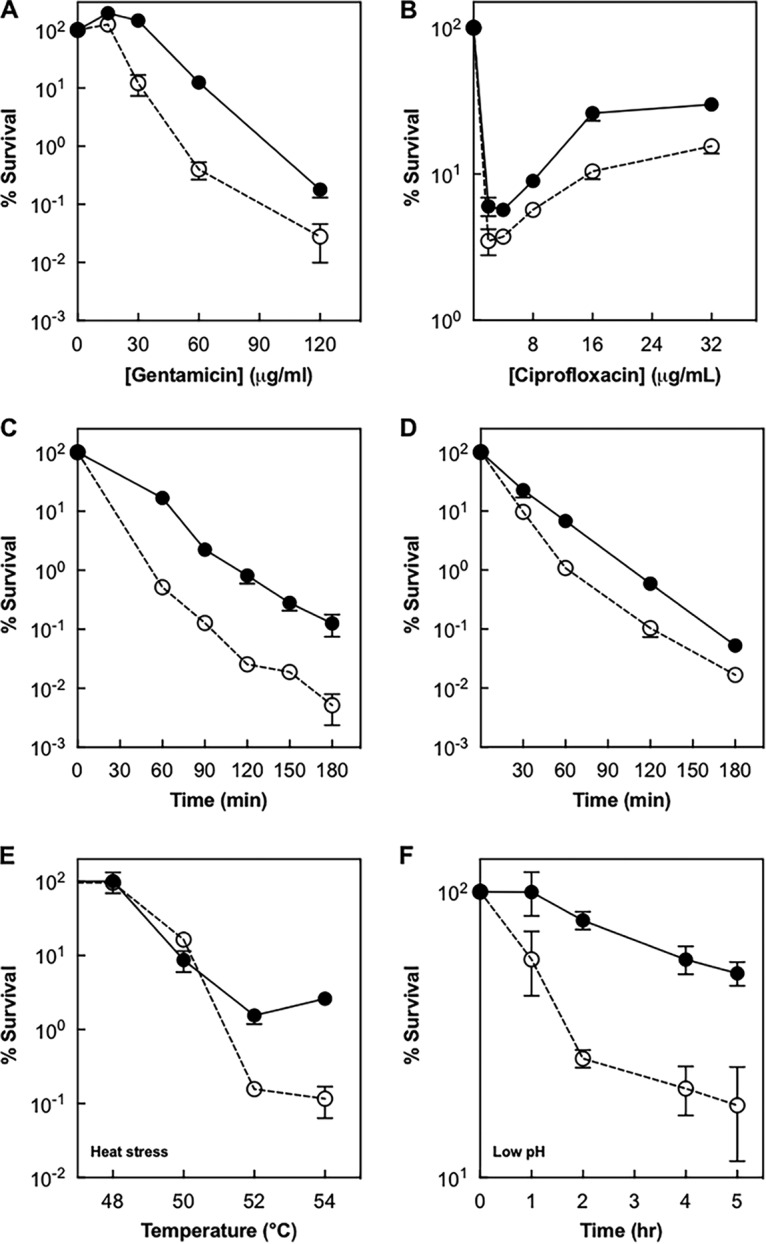
*agr* deficiency increases bacterial survival following exposure to antimicrobial and environmental stresses. Wild-type *S. aureus* Newman (BS12) and the Δ*agr* strain (BS13) were grown to late log phase in TSB and treated with the indicated concentrations of gentamicin for 60 min (A) or with 60 μg/ml of gentamicin for the times indicated (C). Likewise, cultures were treated with indicated concentrations of ciprofloxacin for 60 min (B) or with 10 μg/ml of ciprofloxacin for the times indicated (D). At the end of treatment, aliquots were removed, serially diluted, and plated for determination of viable counts, from which percent survival was calculated relative to a sample taken at the time of drug addition. Similarly, cells grown to late log phase in TSB were incubated at the indicated temperatures for 10 min (E) or in acidic TSB (pH 3.0) for indicated times (F). Symbols: filled circles, Δ*agr* strain; empty circles, wild type. Data represent means from biological replicates ± standard deviations (*n* = 3).

When we compared survival rates of *Δagr* mutant and wild-type cells, we found that the mutation increased survival by about 10-fold for gentamicin and approximately 3-fold for ciprofloxacin when drug concentration was varied (Fig. 1A and B). A similar result was obtained using various treatment times at a fixed drug concentration (Fig. 1C and D). Since the presence of serum may alter antimicrobial activity (43) and *agr* functionality (44), we also examined lethal activity in 20% (vol/vol) human serum, the highest concentration of serum that failed to detectably affect bacterial growth. Inclusion of serum reduced *agr*P3-*blaZ* expression by severalfold (Fig. S1B), but protection conferred by the *agr* deficiency was greater against gentamicin and similar against ciprofloxacin relative to that observed in the absence of serum (Fig. S2A to D). Collectively, these data show that rapid lethal activity responds differently to an *agr* defect than bacteriostatic action, as if wild-type *agr* specifically stimulates a lethal response to the primary damage.

10.1128/mBio.01476-17.2 FIG S2  Serum enhances *agr*-mediated differences in antimicrobial killing. Wild-type *S. aureus* Newman (BS12) and Δ*agr* mutant (BS13) were grown to late log phase in TSB with serum and treated with the indicated concentrations of gentamicin for 60 min (A) or with 60 μg/ml of gentamicin for the times indicated (C). Likewise, cultures were treated with indicated concentrations of ciprofloxacin for 60 min (B) or with 10 μg/ml of ciprofloxacin for the times indicated (D). Symbols: filled circles, Δ*agr* strain; empty circles, wild type. Data represent means of biological replicates ± standard deviations (*n* = 3). Download FIG S2, TIF file, 0.1 MB. Copyright © 2017 Kumar et al. 2017 Kumar et al. This content is distributed under the terms of the Creative Commons Attribution 4.0 International license.

**FIG 2:**
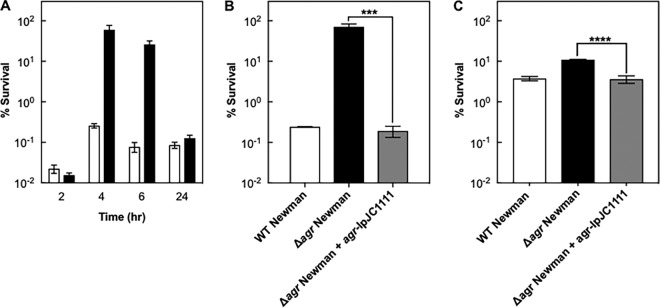
Antimicrobial-mediated killing is growth phase and *agr* specific. Cells were grown in serum for 2, 4, 6, or 24 h as indicated and treated with 15 μg/ml of gentamicin for 60 min (A). To determine whether the observed difference in killing was due to *agr*, wild-type (WT; BS12), Δ*agr* mutant (BS13), and complemented Δ*agr* mutant (BS519) cells were treated with 15 μg/ml of gentamicin (B) or 2.5 μg/ml of ciprofloxacin (C) for 60 min. Survival was determined as described in the legend to Fig. 1. Significance was examined by unpaired two-tailed *t* test (*P* < 0.05). ***, *P* < 0.01; ****, *P* < 0.001. Data represent the means from biological replicates ± standard deviations (*n* = 3).

As expected, *agr*-mediated differences in killing correlated with growth phase, with maximal effects occurring in late exponential phase when *agr* was induced (Fig. 2A). Complementation tests employing chromosomally integrated, wild-type *agr* confirmed that the *agr* deletion was responsible for protection from killing by gentamicin and ciprofloxacin (Fig. 2B and C). A protective effect of the *agr* mutation was also observed when cells were treated with heat or low pH (Fig. 1E and F), suggesting that a destructive, wild-type *agr*-mediated response occurs with a variety of severe stressors.

**FIG 3:**
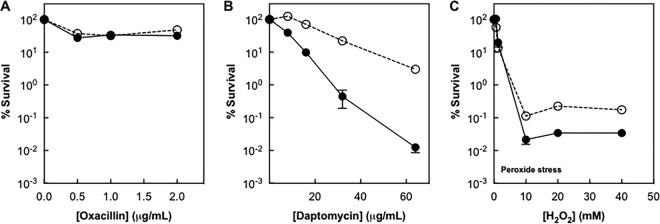
Effect of an *agr* deficiency on the lethal activity of oxacillin, daptomycin, and exogenous H_2_O_2_. Wild-type cells (BS12, empty circles) or *agr*-deficient mutant cells (BS13, filled circles) were grown to late log phase and then treated with the indicated concentrations of oxacillin for 3 h (A), daptomycin for 90 min (B), or H_2_O_2_ for 60 min (C). Survival was determined as described in the legend to Fig. 1. Growth medium was TSB (oxacillin and H_2_O_2_) or Ca^2+^-supplemented Mueller-Hinton broth (daptomycin). Data represent the means from biological replicates ± standard deviations (*n* = 3).

Results with the other two antimicrobials differed from those described above. Killing by oxacillin was unaffected by deletion of *agr* (Fig. 3A). Oxacillin exhibits low to moderate, time-dependent killing. As with killing with other cell wall agents, such as vancomycin, killing by oxacillin likely reflects processes that are different from those occurring with agents that kill more rapidly, such as ciprofloxacin and gentamicin. Daptomycin, a rapid-killing, cell-membrane-targeting agent, showed increased killing in the *agr*-deficient mutant (Fig. 3B). Killing by the environmental stressor hydrogen peroxide was also increased by the *agr* deficiency (Fig. 3C). For these two stressors, wild-type *agr* exhibited a protective role. Thus, inactivation of *agr* protects cells from killing by some types of stress, while it has little effect on or enhances the lethal action of others.

### Effect of *agr* deficiency on response to gentamicin.

**FIG 4:**
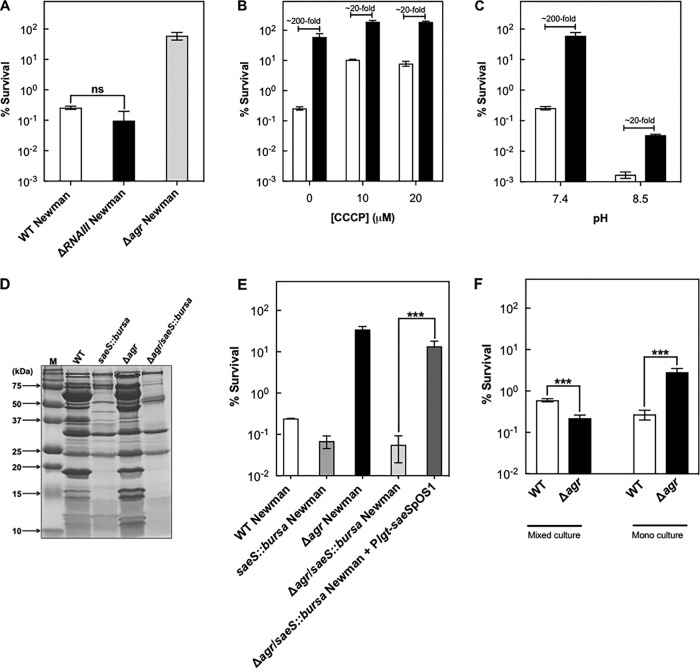
*agrA*-mediated protection from gentamicin-mediated killing is largely independent of PMF. (A) Survival following gentamicin treatment. Late-log-phase cultures were grown in TSB plus 20% (vol/vol) human serum and treated with 15 μg/ml gentamicin for 60 min (wild-type [WT] strain BS12 [white bar], *ΔRNAIII* BS669 [black bar], and *Δagr* mutant BS13 [gray bar]). (B) Effect of pretreatment with CCCP. Cells were grown as described for panel A; they were pretreated with CCCP for 5 min and subsequently with 15 μg/ml of gentamicin for 60 min. (C) Effect of alkaline pH. Cells were grown as described for panel A, late-log-phase cultures were concentrated by centrifugation, and cells were resuspended in serum-containing TSB medium with pH adjusted to 8.0. They were then treated with 15 μg/ml of gentamicin for 60 min. Symbols: white bars, wild-type strain BS12; black bars, *agr* mutant BS13. (D) Effect of *Δagr* or *saeS*::*bursa* single mutation (BS13 and BS984) and double mutation (BS985) on exoproteins. Exoproteins were extracted from late-log-phase cultures as described in Materials and Methods and separated by electrophoresis in a 15% polyacrylamide gel containing SDS, and protein bands were stained with Coomassie blue. Lane M, molecular mass markers. (E) Effect of *Δagr* or *saeS*::*bursa* single mutations and double mutation on survival during treatment with gentamicin. Cells from late-log-phase cultures were treated with 15 μg/ml of gentamicin for 60 min, and then survival was determined. (F) Mixed-culture killing. Wild-type cells (BS12) and an *agr* mutant (BS13) were mixed in equal amounts and grown together in TSB to late log phase; cultures were then treated with 60 μg/ml of gentamicin for 60 min. Percent survival in mixed-culture kill assays was calculated by enumerating survivor colonies grown on sheep blood agar. Unpaired two-tailed *t* test was used to evaluate the significance (*P* < 0.05). ns, not significant (*P* > 0.05); ***, *P* < 0.01. Data represent the means from biological replicates ± standard deviations (*n* = 3).

To better understand the destructive effect of wild-type *agr*, we first asked whether the protective effect of an *agr* deletion against gentamicin-mediated killing acts through RNAIII. We observed no effect (Fig. 4A), indicating that destruction is *agrA* dependent.

Aminoglycosides, such as gentamicin, require the proton motive force (PMF) of the bacterial membrane for penetration into cells (45). The PMF consists of a transmembrane pH gradient and a transmembrane electrical potential. Thus, inhibitors that eliminate the proton gradient, such as carbonyl cyanide *m*-chlorophenylhydrazone (CCCP), inhibit aminoglycoside uptake and thereby activity (46), while increasing the pH of the medium enhances gentamicin uptake (47). When CCCP was used with gentamicin, a protective effect was still observed with the *Δagr* mutant, and the effect of CCCP was muted (Fig. 4B). When pH was raised, gentamicin became more lethal; the *agr* deficiency remained protective, but less so (Fig. 4C). These partial effects of an *agr* deficiency suggested that wild-type *agr* stimulates antimicrobial lethality for gentamicin through both PMF-dependent and PMF-independent pathways.

*S. aureus* Newman, the strain employed to generate many of the results described above, has a naturally occurring upregulating mutation in the two-component signaling system *sae*. Unlike *agr*, *sae* senses environmental signals (48), rather than a quorum-sensing peptide, to tailor the production of *S. aureus* virulence factors. The upregulating mutation in *sae* results in constitutive activation of numerous genes that contribute to the exoproteome of Newman strains, even when *agr* is absent (49, 50). To test the possibility that *sae* lies on the pathway leading to protection from the lethal activity of gentamicin, we performed killing assays employing an engineered strain deficient in both *agr* and *saeS* (BS985). The double mutant demonstrated an almost complete lack of exoprotein secretion (Fig. 4D), and it showed survival of gentamicin treatment comparable to that of wild-type cells (Fig. 4E). Thus, the *saeS* deficiency reversed the protective effect of *Δagr*. As expected, a *sae*-complemented strain was killed to the same extent as the *Δagr* strain (Fig. 4E). These data suggest that exoproteins are a source of protection from gentamicin-mediated killing of *S. aureus* afforded by *agr*-inactivating mutations. To explore this possibility, we examined whether differences seen between *agr*-positive and *agr*-defective strains in monoculture are eliminated in coculture. In coculture with equal starting inocula, the difference between the wild-type and *agr* mutant strains was much smaller with respect to killing by gentamicin, consistent with complementation in *trans* through a shared, extracellular factor (Fig. 4F).

### *S. aureus* strains vary in protection from lethal stress by an *agr* deficiency.

**FIG 5:**
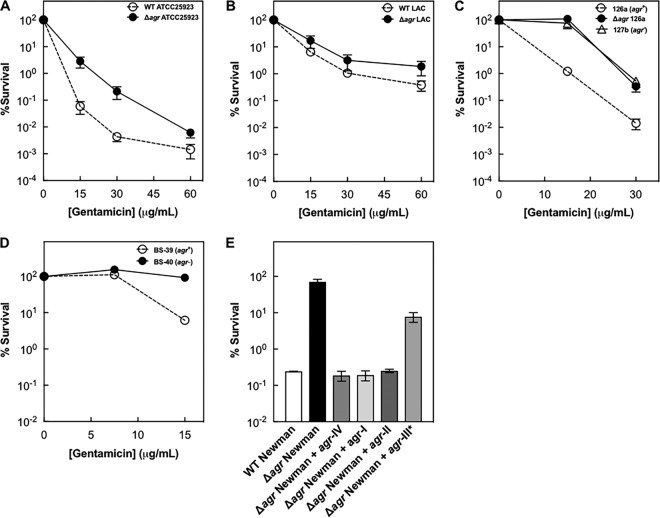
*agr*-mediated phenotypes among diverse *S. aureus* strains. (A to D) Wild-type (WT) laboratory strains ATCC 25923 (BS902) (A) and LAC (BS819) (B) and clinical isolates 127b (C) and BS40 (D) with naturally occurring *agr* mutations (indicated in panels) were grown to late log phase and treated with indicated concentrations of gentamicin for 60 min. Symbols: open circles, wild-type cells; filled symbols, *Δagr* mutants. (E) Effect of *agr* group-specific differences on survival with gentamicin. Newman and *Δagr* strains and isogenic variants with the indicated *agr* allele (I to IV; BS519 to BS522) were grown to late log phase in TSB and treated with 15 μg/ml of gentamicin for 60 min. The asterisk denotes the strain containing the *agr*-III allele (BS519), which has a mutation in *agrC* that attenuates *agr* function (3) (see text). Data represent the means from biological replicates ± standard deviations (*n* = 3).

We next examined the effect of *agr* functionality with the methicillin-susceptible strain ATCC 25923 and with the prototype community-acquired methicillin-resistant strain LAC. The protective effect of an *agr* deletion on killing by gentamicin was observed with strain ATCC 25923 but was marginal in LAC (Fig. 5A and B). Thus, the effect of an *agr* deficiency on the response to lethal stress is strain dependent, but it is not lineage specific.

Differences observed in assays utilizing laboratory-constructed mutants may be obscured during infection by changes elsewhere in the genome. Accordingly, we assayed clinical *agr*-defective strains using a small set of genotypically diverse *agr*-defective MRSA clones initially obtained from mixed cultures containing *agr*-positive and *agr*-negative cells (51). Strains were previously genotyped to confirm that variants within a single specimen were otherwise isogenic (51). That work also traced the basis of *agr* dysfunction to inactivating mutations in *agrA* or *agrC*. Of the 4 clinical isolates in our collection that were susceptible to gentamicin, 2 genotypically distinct clones (see Table S1 in the supplemental material) exhibited 10-fold protection from killing by gentamicin (Fig. 5C and D). Thus, the data are consistent with data from laboratory strains indicating that *agr* inactivation reveals cryptic genetic variation among strains in their response to lethal stress. Moreover, variation in intrinsic (wild-type) tolerance was also observed. The mechanisms underlying strain-dependent differences in *agr*-mediated and intrinsic tolerance to lethal stress are unknown. Future work will investigate to what extent they are stress specific.

Previous work shows allelic variation in the *S. aureus agr* genes, identified as four specificity groups based on induction timing and strength. Induction is the earliest and strongest with *agr*-IV and -I, *agr*-II is intermediate, and induction with *agr*-III is delayed and weak (38). Weak *agr*-III induction levels result from a single-nucleotide polymorphism that changes amino acid 55 of AgrC (G55R) (10). This substitution is found in hospital-associated MRSA (HA-MRSA) clonal complex 30 lineage clones associated with poor outcome in bacteremic patients (16, 52). When we examined group-specific differences in *agr* autoinduction and virulence gene regulation using previously characterized isogenic variants of strain Newman (28), each harboring an *S. aureus agr* allele, we found an *agr*-III-specific attenuation of protection to gentamicin (Fig. 5E). Thus, partial loss-of-function mutations of *agr* may tune levels of signaling to balance virulence and antimicrobial tolerance.

### Effect of *agr* deficiency on stress response to ciprofloxacin.

**FIG 6:**
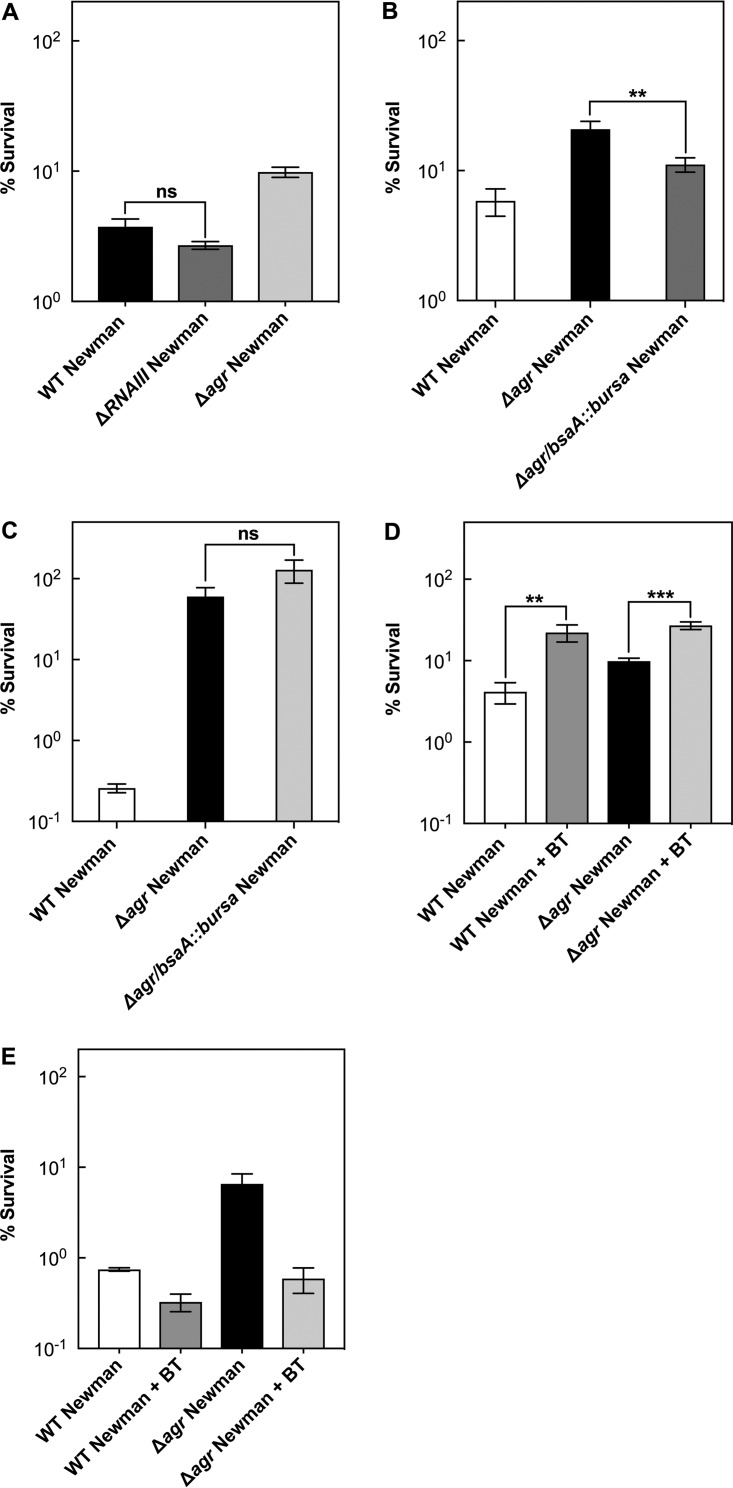
*agrA*-mediated protection from ciprofloxacin-mediated killing involves ROS-dependent and ROS-independent pathways. (A to C) Effect of RNAIII and *bsaA* on survival during treatment with ciprofloxacin or gentamicin. Wild-type (WT) strain Newman (BS12), *Δagr* mutant (BS13), and *ΔRNAIII* mutant (BS669) (A) or wild-type strain Newman, *Δagr* mutant, and *Δagr ΔbsaA* double mutant (BS982) (B) were grown to late log phase and treated with 2.5 μg/ml of ciprofloxacin. Wild-type strain Newman, the *Δagr* mutant, and the *Δagr bsaA*::*bursa* double mutant were treated with 60 μg/ml gentamicin (C). (D and E) Effect of ROS quenchers on killing. Cells were grown to late log phase in TSB and treated with 10 μg/ml of ciprofloxacin (D) or 60 μg/ml of gentamicin (E) in the presence of 2,2′-bipyridyl and thiourea (BT), each at 0.5× MIC, for 60 min. Significance was determined by unpaired two-tailed *t* test (*P* < 0.05). ns, not significant; **, *P* < 0.05; ***, *P* < 0.01. Data represent the means from biological replicates ± standard deviations (*n* = 3).

As with gentamicin, the protective effect of an *agr* deletion against ciprofloxacin-mediated killing does not act through RNAIII—the Δ*RNAIII* mutant demonstrated no effect on killing for ciprofloxacin, indicating that protection is *agrA* dependent (Fig. 6A).

The DNA-binding domain of AgrA contains an intramolecular disulfide switch as part of an oxidation-sensing mechanism. Oxidation leads to dissociation of AgrA from DNA, which prevents AgrA-mediated downregulation of glutathione peroxidase (BsaA), an enzyme that detoxifies ROS (53). Accordingly, inactivating mutations in *agr* elevate the expression of *bsaA*, which is expected to reduce oxidation-mediated lethality arising from treatment with antimicrobials such as fluoroquinolones (33). A *Δagr bsaA*::*bursa* double mutation lowered survival following treatment with ciprofloxacin but not with gentamicin (Fig. 6B and C), indicating that *agr* acts differently on the effects of the two drugs.

Since *bsaA* is involved in detoxification of ROS, we tested for elimination of Δ*agr*-mediated protection by an ROS scavenger and iron chelator. When wild-type and *Δagr* mutant cells were pretreated with subinhibitory (0.5× MIC) concentrations of thiourea plus 2,2′-bipyridyl to block hydroxyl radical accumulation, the lethal action of ciprofloxacin, but not gentamicin, was reduced in both strains, and the *Δagr*-mediated protection for ciprofloxacin was eliminated (Fig. 6D and E). These data indicate that wild-type *agr* stimulates ciprofloxacin lethality largely through an ROS-dependent pathway. These data are consistent with the observation that ciprofloxacin MBCs were identical for wild-type and *Δagr* strains (1 μg/ml): MBC reflects killing extent, while ROS-mediated effects are seen as rate changes (32, 33). The observed lack of an effect of *Δagr* on peroxide-mediated killing is consistent with previous work indicating that the action of exogenous hydrogen peroxide overshadows endogenous-ROS-mediated effects (54).

Overall, *agr* deficiency-dependent escape from lethal stress is common among *S. aureus* strains and correlates with reduction in the activity of some antimicrobials (gentamicin) and interference in the lethal response to others (ciprofloxacin). Thus, representative stressors illustrate how mutations in *agr* reveal a general mechanism of adaptive evolution through attenuation of lethal stress. Complexity was uncovered by daptomycin having the opposite effect, as described below in the Discussion.

## DISCUSSION

The work described above addresses the general issue of adaptive leaps made by bacterial pathogens, using as an example the paradoxical finding that defects in the *agr* virulence regulon are associated with poor patient outcome from staphylococcal infection, particularly during antimicrobial treatment (27, 28). The major observation is that wild-type *agr* stimulates the lethal action of several stressors, including gentamicin and ciprofloxacin; thus, defective mutants will tend to persist under stressful conditions rather than being killed by stressors that may include synthetic antimicrobials and host defenses such as neutrophil-generated ROS. In the case of gentamicin, *agr*-mediated stimulation of lethal activity relies on the bacterial production of exoproteins; for ciprofloxacin, *agr* normally downregulates a protein that protects from ROS. How *agr* stimulates killing by heat and low pH is not yet known. The lethal action of these diverse stressors may apply to many other challenges experienced by *S. aureus* during persistent infection and thereby help explain the selective enrichment of *agr*-deficient mutants. Since stimulation of killing by *agr* is not universal, as shown by daptomycin being more lethal with the *agr* mutant, determining whether *agr* has a positive or negative effect on the lethality of a particular stressor will be important for combining anti-Agr agents with antimicrobials.

A striking observation was the absence of an *agr* effect on bacteriostatic activity. Previous distinctions between bacteriostatic and lethal activity with fluoroquinolones (55) led to the idea that some forms of lethal activity, in particular those involving ROS, are a cellular response to a primary lesion (34). The current work strongly supports separation of bacteriostatic and bactericidal effects, thereby emphasizing the need to normalize survival data to MIC when considering how stress kills bacterial cells (32). Our conclusion that lethal activity exerts selective pressure independently of bacteriostatic activity also emphasizes the importance of considering antimicrobial lethality during bacterial infection (current treatment, diagnosis, and surveillance are based largely on bacteriostatic activity [MIC]).

The ability of bacteria to survive lethal stress that still blocks growth is a form of tolerance: paradoxical enrichment of *agr*-deficient mutants is a clinical example of tolerance that would not be detected by standard susceptibility testing for resistance. Our results support the growing body of evidence that mutations in global regulators constitute a prominent mechanism underlying tolerance (56–60). From a clinical perspective, tolerance to severe stress presents a major challenge: in contrast to the specificity of resistance, tolerance can confer a survival advantage against a broad spectrum of selective pressures that ultimately lead to antimicrobial resistance (59) and to altered host-pathogen interactions that favor persistent infection. Thus, understanding tolerance is critical for addressing the decreasing efficacy of antibiotics.

Within our sample of stressors, daptomycin was unusual in exhibiting greater lethality with the *agr*-deficient mutant. Test conditions are important, as indicated by consideration of previous work in which the opposite result was obtained with nongrowing *S. aureus* in deep stationary phase, long after induction of *agr* and expression of *agr* transcripts (61). Daptomycin causes the release of membrane phospholipids that bind to and inactivate the antibiotic (61); *agrA* triggers secretion of phenol-soluble modulins (PSMs) that bind to phospholipids and prevent daptomycin inactivation. Our experiments were performed in late exponential phase when PSM levels may be lower and less protective (18). The complex relationship between daptomycin lethality, *agr* status, and bacterial physiological state illustrates the importance of understanding *agr* biology before applying novel therapies that target *agr* (62).

Secreted factors that bind to drugs or block their uptake are expected to affect the MIC. Since no difference in MIC was observed for *agr* alleles (Table 1) (61), the protective mechanism induced by daptomycin, described by Pader et al. (61), likely involves cell damage and release of phospholipids occurring at drug concentrations above the MIC. As with daptomycin, gentamicin interacts strongly with anionic sites in the plasma membrane and in particular phospholipids. We reason that concentrations above MIC are required to trigger leakage of bacterial components, explaining the lack of *agr*-mediated perturbation of MIC for gentamicin.

Ciprofloxacin-mediated killing merits additional comment because a role for ROS still remains controversial (63–65). *agr* normally downregulates *bsaA*, a gene encoding glutathione peroxidase, which detoxifies ROS (53). The *Δagr* defect allows expression of a protective protein, thereby explaining the drop in ciprofloxacin-mediated killing. Thus, the present work is most readily explained by a contribution of ROS to killing by quinolones and helps resolve a controversy (31, 32, 35). Indeed, with *S. aureus* ciprofloxacin is more likely to exhibit ROS-mediated lethality than are more potent fluoroquinolones that tend to kill by an ROS-independent mechanism, as deduced from studies of *Escherichia coli* (66, 67).

Previous reports indicate that inactivation of RNAIII is associated with a growth advantage for Δ*agr* mutants in the presence of subinhibitory concentrations of several antibiotics (ciprofloxacin, mupirocin, and rifampin) (25). These data, plus the present work, lead to the conclusion that two distinct subsets of *agr* antimicrobial fitness exist: an RNAIII-independent one that impacts antimicrobial lethality and an RNAIII-dependent form that controls antimicrobial-associated fitness for growth. The mechanism underlying *agr* dysfunction among strains derived from clinical isolates is almost always traced to inactivating mutations in *agrC* and *agrA*, the sensor component and response regulator, respectively, of the *agr* system (14–17). Since selection for *agr*-defective strains occurs in mixtures with *agr*-positive parental strains, inactivation of *agrD* or *agrB* does not silence *agr* (autoinducing peptide is produced in *trans* by the *agr*-positive strain). However, this scenario does not explain why RNAIII is not targeted by selection for loss of *agr* function. Identification of the role of *agrA* in protection from the lethal response to antimicrobial-mediated stress resolves the dilemma, since inactivating mutations in *agrCA* will inactivate both *agr*P2-*agrA* and *agr*P3-RNAIII operons.

In summary, comparison of clinical strains entering hospitals with those emerging from patients provides insight into how infection remodels pathogens with respect to a major regulator. Additional lethality screening is needed to determine the frequency and specificity with which *agr* inactivation results in tolerance to specific stresses among clinical *agr*-defective mutants. We expect that additional lethality screening will identify other bacterial regulators having activities that can be either destructive or protective, depending on the type and level of lethal stress. Understanding the basis for such antimicrobial tolerance can be clinically significant when it informs efforts to personalize antimicrobial management through strain-specific pathogen characteristics. For example, use of anti-*agr* agents or therapeutic vaccines (62) may be ill advised for applications in which the absence of *agr* reduces antimicrobial lethality. Identifying other adaptations that erode the lethal activities of antimicrobials could lead to novel strategies for selectively bolstering antimicrobial effectiveness (68–70).

## MATERIALS AND METHODS

### Bacterial strains, plasmids, and growth conditions.

*S. aureus* strains and plasmids used in the study are described in Table S1 in the supplemental material. Cells were cultured in tryptic soy broth (TSB) with constant aeration (rotary shaking at 250 rpm) or on tryptic soy agar (TSA) plates. In some cases, TSB was supplemented with 20% (vol/vol) human serum. Incubation was at 37°C. Phages 80α and Φ11 were used to transduce marker-disrupted alleles (71); transductants were selected on TSA plates containing the appropriate antimicrobial.

10.1128/mBio.01476-17.3 TABLE S1  Strains. Download TABLE S1, DOCX file, 0.03 MB. Copyright © 2017 Kumar et al. 2017 Kumar et al. This content is distributed under the terms of the Creative Commons Attribution 4.0 International license.

### Antimicrobials and chemicals.

Antimicrobials and off-the-clot human serum were obtained from Sigma-Aldrich (St. Louis, MO) and SeraCare (Milford, MA), respectively. Chemicals and reagents were obtained from Sigma-Aldrich and Fisher (Fair Lawn, NJ).

### Construction of mutants.

*S. aureus* Newman *agr*::*tet* and *RNAIII*::*cd* were generated by transducing the disrupted alleles from RN6911 and BS640, respectively, using phage 80α. The Δ*agr bsaA*::*bursa* double mutant (strain BS982) was generated by transducing *bsaA*::*bursa*, obtained from the University of Nebraska transposon mutant (ΦNE) library, into *agr*::*tet* Newman using phage 80α. An *S. aureus* Newman Δagr *saeS*::*bursa* double mutant was obtained by moving *sae*S::*bursa* from strain VJT12.22 (51) to *agr*::*tet* Newman using phage 80α-mediated transduction. For *saeS* complementation, plasmid P_lgt_-*saeS*pOS1, expressing *saeS* under the control of the constitutive promoter *hprK*, was introduced into the double mutant strain Newman *agr*::*tet saeS*::*bursa* by bacterial transduction (51).

### Reporter assays.

We employed an *agr*P3-*blaZ* reporter cassette integrated into the *S. aureus* chromosome at the SaPI1 *attC* site (72). Overnight cultures were diluted to an optical density at 600 nm (OD_600_) of 0.05 in TSB with or without 20% (vol/vol) human serum and incubated at 37°C with shaking. Cultures were collected at various times; normalized β-lactamase activity (*V*_max_/OD_600_) was determined using the nitrocefin method as described previously (38). Briefly, 50 μl cells was mixed with 50 μl of nitrocefin solution (119 μg/ml prepared in 100 mM sodium phosphate buffer, pH 5.8); OD_490_ and OD_600_ were measured using a Synergy H1 hybrid microplate reader (BioTek).

### Exoprotein analysis.

Exoproteins were extracted as described previously (26). Briefly, cells were grown overnight in hydrolysate-yeast extract-containing medium (CCY) (3% [wt/vol] yeast extract, 2% Bacto Casamino Acids, 2.3% sodium pyruvate, 0.63% Na_2_HPO_4_, and 0.041% KH_2_PO_4_ [pH 6.7]). Overnight cultures were diluted and grown to late log phase (~5 h) in fresh CCY, and 1.5-ml aliquots were centrifuged to remove bacteria. Culture supernatants were treated with an equal volume of ice-cold 20% trichloroacetic acid, and the precipitate was collected by centrifugation. The precipitated proteins were separated and analyzed by sodium dodecyl sulfate-polyacrylamide gel electrophoresis (73).

### Susceptibility and survival measurements.

Inhibition of growth (MIC values, Table 1) was determined by agar or broth dilution. For the latter, about 10^5^ cells were applied to a series of broth cultures containing antimicrobials at various concentrations (2-fold dilutions). Turbid growth and optical density (OD_600_) were detected after 1 day. The MIC was taken as the minimal concentration that blocked growth of liquid cultures.

To measure lethal action, overnight cultures were diluted 50-fold in TSB or TSB plus serum and grown with shaking to late log phase, a condition in which *agr* is maximally activated. Cultures (~3 × 10^8^ CFU/ml) were exposed to antimicrobials under aerobic conditions, diluted in drug-free medium, plated on drug-free agar, and incubated overnight at 37°C. Percent survival was estimated by colony formation relative to that of an untreated control sampled at the time of antimicrobial addition. To measure the effect of alkaline pH on gentamicin-mediated lethality, the pH of serum was adjusted with 6 N NaOH to 8.5. CCCP was added to cultures 5 min prior to the addition of gentamicin. To test the effect of ROS quenchers on gentamicin- and ciprofloxacin-mediated lethality, cultures grown in TSB were treated with 0.5× MIC of 2,2′-bipyridyl and thiourea 5 min prior to the addition of the antimicrobial. Since daptomycin requires Ca^2+^ for activity, late-log-phase cultures were supplemented with 50 μg/ml Ca^2+^ and 25 μg/ml Mg^2+^ when treated with various concentrations of daptomycin for 90 min in Mueller-Hinton broth. To measure the lethal effects of high-temperature stress, cells were grown in TSB to ~3 × 10^8^ CFU/ml, incubated at various temperatures for 10 min in a PCR thermocycler (Eppendorf, Hamburg, Germany), serially diluted, and plated on drug-free agar for determination of viable colony numbers. To measure the lethal effect of low pH, cells from late-log-phase cultures were concentrated by centrifugation, resuspended in TSB adjusted to various values of pH with HCl, and incubated at 37°C under aerobic conditions for various times. For H_2_O_2_ treatment, late-log-phase cultures (~10^7^ CFU/ml) were treated with various concentrations of peroxide for 1 h at 37°C under aerobic conditions. All experiments were repeated at least three times; similar results were obtained from the biological replicates.

### Statistical analysis.

For killing assays, comparisons were made using an unpaired two-tailed *t* test (*P* < 0.05). *P* values of <0.05 were considered statistically significant.
