# Supplementary figures and images for: Tuning of the Lethal Response to Multiple Stressors with a Single-Site Mutation during Clinical Infection by Staphylococcus aureus
Source: mBio. 2017 Oct 24;8(5):e01476-17. doi: 10.1128/mBio.01476-17 (PMC5654930; doi:10.1128/mBio.01476-17)

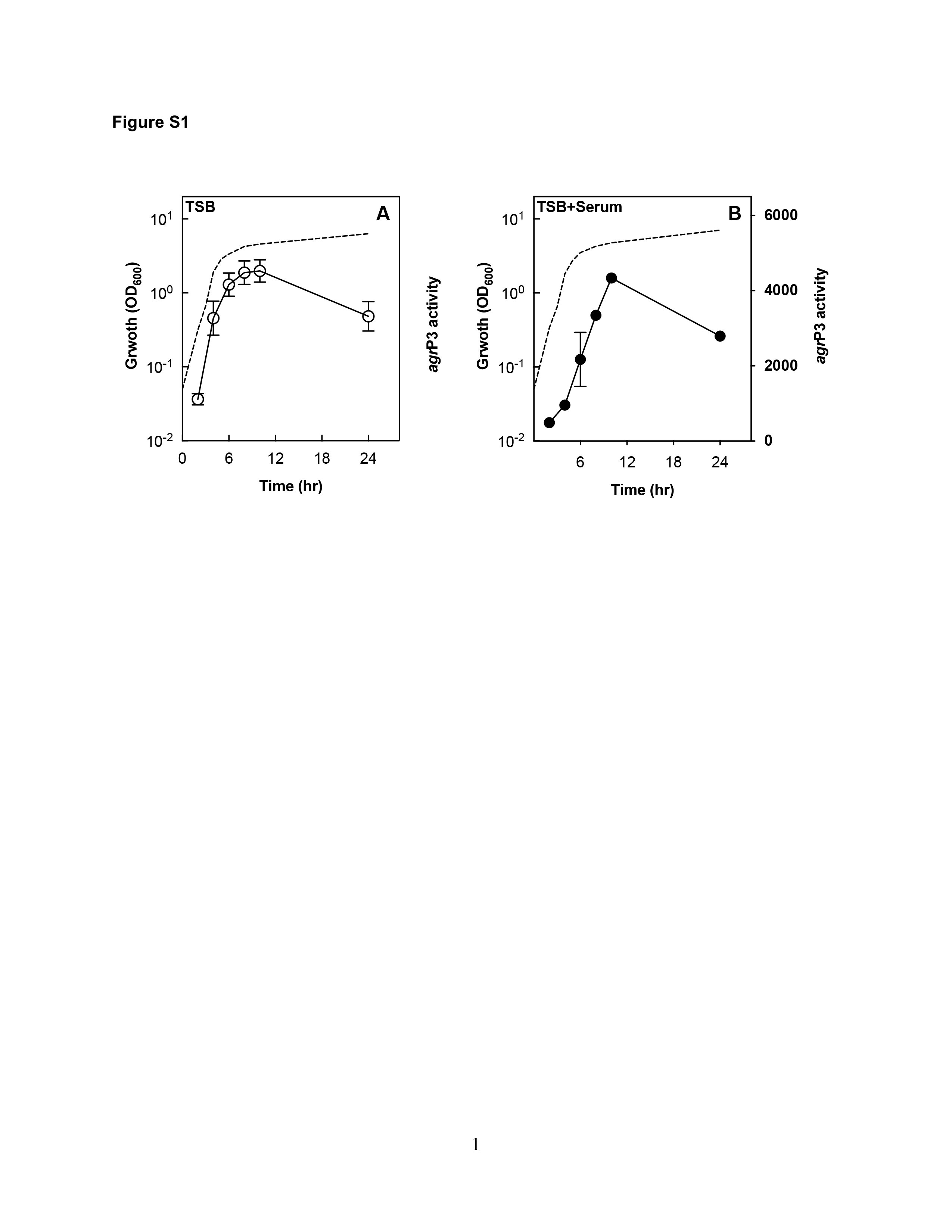

Supplement: FIG S1 [file mbo005173552sf1.tif]

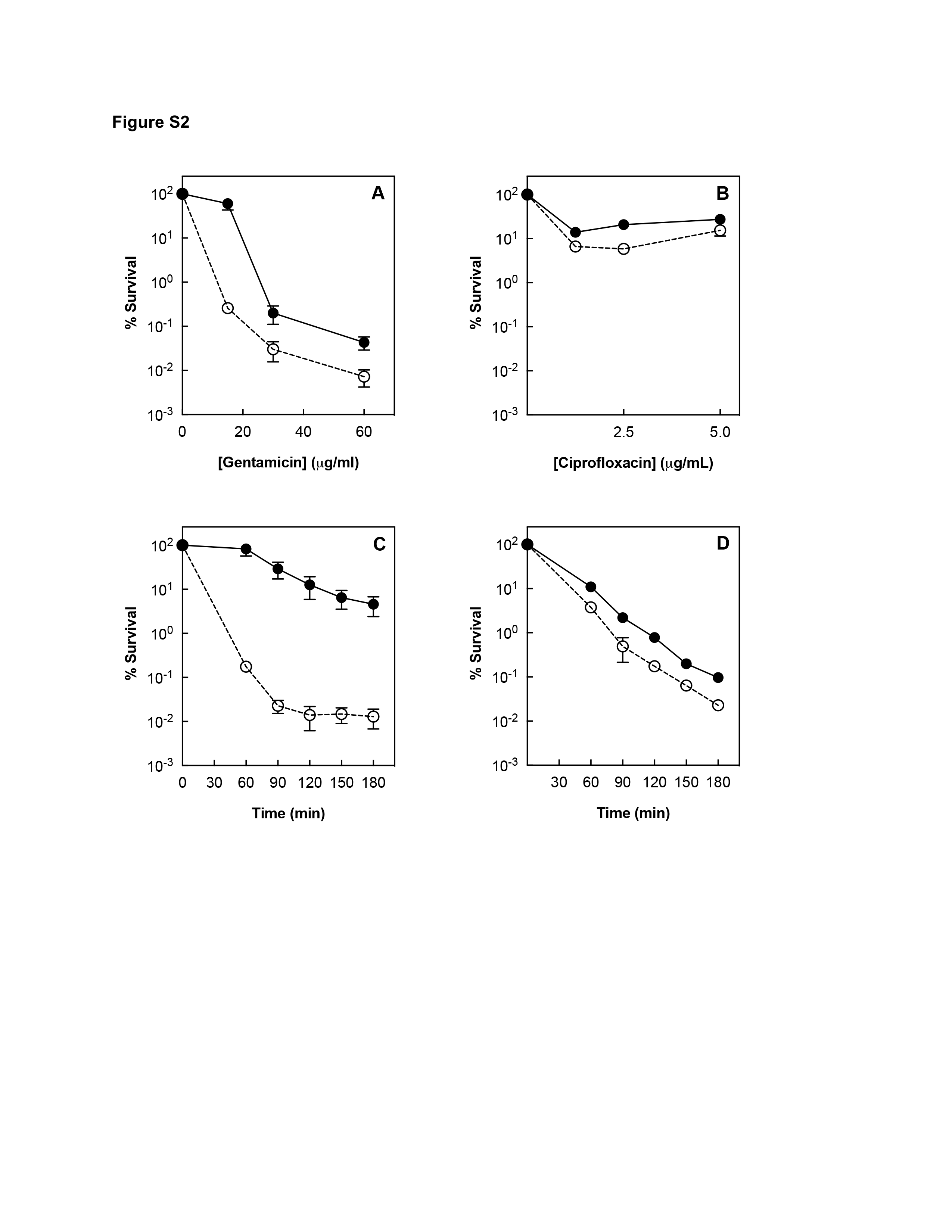

Supplement: FIG S2 [file mbo005173552sf2.tif]
