# Supplementary material for: Tuning of the Lethal Response to Multiple Stressors with a Single-Site Mutation during Clinical Infection by Staphylococcus aureus
Source: mBio. 2017 Oct 24;8(5):e01476-17. doi: 10.1128/mBio.01476-17 (PMC5654930; doi:10.1128/mBio.01476-17)
Supplement: TABLE S1 [file mbo005173552st1.docx]

**Supplementary Data**

**Table S1. Strains**

| **Strain** | | **Background** | | **Relevant Genotype or Description** | | **Reference or Source** | |
| --- | --- | --- | --- | --- | --- | --- | --- |
| BS12 | | Newman | | Wild-type (*agr* group I, CC8) | | [1] | |
| BS13 | | *Δagr* Newman | | *agr::tet,* transductant of RN6911 | | [2] | |
| BS519 | | *Δagr* + *agr*-IpJC1111 Newman | | HF6122 (*agr*::*tetM*, Newman) congenic *agr* I-IV variants | | [3] | |
| BS520 | | *Δagr* + *agr*-IIpJC1111 Newman | |  |  |  |  |
| BS521 | | *Δagr* + *agr*-IIIpJC1111 Newman | |  |  |  |  |
| BS522 | | *Δagr* + *agr*-IVpJC1111 Newman | |  |  |  |  |
| BS669 | | *ΔRNAIII* Newman | | *RNAIII::cad;* transductant of BS-640 | | [4] | |
| BS982 | | *Δagr*/*bsaA::bursa* Newman | | *bsaA::bursa;* transductant of Nebraska library clone | | [5] | |
| BS983 | | *agrP3-blaZ*pJC1111 Newman | | *agrP3*-β-lactamase reporter; SaPI1 *att_c_*::*agrP3-blaZ*pJC1648 (Cd) | | This study | |
| BS984 | | *saeS::bursa* Newman | | *saeS::bursa;* transductant of Nebraska library clone | | [5] | |
| BS985 | | *Δagr*/*saeS::bursa* Newman | | *agr::tet*/*saeS::bursa* Newman; transduction of *saeS::bursa* to BS13 | | [6] | |
| BS986 | | *Δagr*/*saeS::bursa* + P*lgt*-*saeS*pOS1 Newman | | P*lgt*-*saeS*pOS1; transductant of *agr::tet*/*saeS::bursa* Newman | | [6], [7] | |
| BS902 | | ATCC25923 | | Wild-type (*agr* group III, CC30) | | ATCC | |
| BS987 | | *Δagr* ATCC25923 | | *agr::cad;* transductant of JCSA456 | | This Study | |
| BS39 | | BS39 clinical strain | | *agr* (+) clinical strain (CC45) | | [6] | |
| BS40 | | BS40 clinical strain | | *agr* (-) clinical strain (CC45) | | [6] | |
| BS988 | | *126a* | | *agr* (+) clinical strain (CC5) | | [6] | |
| BS989 | | *127b* | | *agr* (-) clinical strain (CC5) | | [6] | |
| BS990 | | *Δagr* 126a | | *agr::tet,* transductant of RN6911 | | [2] | |
| BS819 | | LAC | | Wild-type (*agr* group-I, CC8) | | [8] | |
| BS820 | | *Δagr* LAC | | *agr::erm,* transductant of JCSA458 | | This Study | |

Abbreviations: CC, clonal complex.

**References for Supplementary Table S1**

1. **Novick RP, Ross HF, Projan SJ, Kornblum J, Kreiswirth B, Moghazeh S.** 1993. Synthesis of staphylococcal virulence factors is controlled by a regulatory RNA molecule. EMBO J **12:**3967-3975.
2. **Bae T, Banger AK, Wallace A, Glass EM, Aslund F, Schneewind O, Missiakas DM.** 2004. *Staphylococcus aureus* virulence genes identified by bursa aurealis mutagenesis and nematode killing. Proc Natl Acad Sci U S A **101:**12312-12317.
3. **Geisinger E, Chen J, Novick RP.** 2012. Allele-dependent differences in quorum-sensing dynamics result in variant expression of virulence genes in *Staphylococcus aureus*. J Bacteriol **194:**2854-2864.
4. **Wilde AD, Snyder DJ, Putnam NE, Valentino MD, Hammer ND, Lonergan ZR, Hinger SA, Aysanoa EE, Blanchard C, Dunman PM, Wasserman GA, Chen J, Shopsin B, Gilmore MS, Skaar EP, Cassat JE.** 2015. Bacterial hypoxic responses revealed as critical determinants of the host-pathogen outcome by TnSeq analysis of *Staphylococcus aureus* invasive infection. PLoS Pathog **11:**e1005341.
5. **Fey PD, Endres JL, Yajjala VK, Widhelm TJ, Boissy RJ, Bose JL, Bayles KW.** 2013. A genetic resource for rapid and comprehensive phenotype screening of nonessential *Staphylococcus aureus* genes. mBio **4:**e00537-00512.
6. **Benson MA, Lilo S, Wasserman GA, Thoendel M, Smith A, Horswill AR, Fraser J, Novick RP, Shopsin B, Torres VJ.** 2011. *Staphylococcus aureus* regulates the expression and production of the staphylococcal superantigen-like secreted proteins in a Rot-dependent manner. Mol Microbiol **81:**659-675.
7. **Benson MA, Lilo S, Nygaard T, Voyich JM, Torres VJ.** 2012. Rot and SaeRS cooperate to activate expression of the staphylococcal superantigen-like exoproteins. J Bacteriol **194:**4355-4365.
8. **Kennedy AD, Otto M, Braughton KR, Whitney AR, Chen L, Mathema B, Mediavilla JR, Byrne KA, Parkins LD, Tenover FC, Kreiswirth BN, Musser JM, DeLeo FR.** 2008. Epidemic community-associated methicillin-resistant *Staphylococcus aureus*: recent clonal expansion and diversification. Proc Natl Acad Sci U S A **105:**1327-1332.
